# A Qualitative Analysis of Dietary Practices and Motivators Among Older Adults Living With Parkinson Disease

**DOI:** 10.1093/geroni/igab046.3552

**Published:** 2021-12-17

**Authors:** Christine Ferguson, Jeannine Lawrence, Joy Douglas, Seung Jung, Anne Halli-Tierney, Chuong Bui, Kyndal Oden, Amy Ellis

**Affiliations:** 1 University of Alabama at Birmingham, Tuscaloosa, Alabama, United States; 2 University of Alabama, Tuscaloosa, Alabama, United States; 3 The University of Alabama, Tuscaloosa, Alabama, United States; 4 University of Alabama, University of Alabama, Alabama, United States

## Abstract

Research supports the role of diet in the onset and progression of Parkinson disease (PD); however, there is no specific dietary pattern recommended for PD. This is partially due to a paucity of in-depth data on the dietary practices of this population. Therefore, the purpose of this study was to qualitatively explore the dietary practices and motivators of older adults with PD. Eleven dyadic semi-structured interviews with older adults and their care-partners were conducted via Zoom about their dietary practices and motivators. Interviews were audio-recorded and transcribed verbatim, and data were thematically analyzed using NVivo 12 software. The following themes were identified: 1) Intentionally making healthier choices on a regular basis; 2) Following a specific dietary pattern; for example, the Mediterranean diet, ketogenic diet, vegetarian diet, and/or intermittent fasting; 3) Limiting or avoiding certain foods, food components, and/or food groups, such as dairy, gluten, sugar, animal meat, and/or alcohol; 4) Purchasing or growing organic produce; and 5) Adjusting the timing of their protein intake to their medications. Their PD diagnosis and symptoms were reported as the primary motivators for following their respective diets. Overall, older adults with PD may be motivated by their diagnosis to modify their dietary intake; however, there are a variety of patterns or restrictions they may be adhering to. These results support the need for a consensus on the dietary recommendations for this patient population.

